# Was ist neu im Management der peripheren arteriellen Verschlusskrankheit und der aortalen Erkrankungen? Highlights der ESC-Leitlinien 2024

**DOI:** 10.1007/s00059-024-05286-7

**Published:** 2024-11-26

**Authors:** Heike Schulze-Bauer, Moritz Staudacher, Sabine Steiner, Oliver Schlager

**Affiliations:** Klinische Abteilung für Angiologie, Universitätsklinik für Innere Medizin II, Medizinische Universität Wien, Währinger Gürtel 18–20, 1090 Wien, Österreich

**Keywords:** Gefäßmedizin, Arterielles Gefäßsystem, Aorta, Ganzheitlicher Ansatz, Leitlinie, Vascular medicine, Arterial vascular system, Aorta, Holistic approach, Guideline

## Abstract

Die im September 2024 veröffentlichten Leitlinien der European Society of Cardiology (ESC) zur peripheren arteriellen Verschlusskrankheit (pAVK) und zu aortalen Erkrankungen bilden erstmals Empfehlungen für beide Krankheitsbilder in einem gemeinsamen Leitliniendokument ab. Die Zusammenführung von pAVK- und Aortenleitlinien folgt einem ganzheitlichen Ansatz, der die Gesamtheit des arteriellen Gefäßsystems unterstreicht. Dieses Ziel wird durch eine eigens eingebrachte Empfehlung unterstrichen, bei Patienten mit Gefäßerkrankungen die Gesamtheit des Kreislaufsystems zu berücksichtigen. Schwerpunkt im aktuellen ESC-Leitlinien-Dokument ist das multidisziplinäre patientenzentrierte Management von pAVK und Aortenerkrankungen, wobei die Prävention und die Nachsorge nach therapeutischen Interventionen hervorgehoben werden. Insbesondere werden bei der pAVK das Gehtraining und das Prozedere bei chronischen Wunden sowie bei Aortenerkrankungen die Risikostratifizierung und hereditäre Aortenerkrankungen behandelt.

## Abstract (en)

The European Society of Cardiology (ESC) guidelines on peripheral arterial disease (PAD) and diseases of the aorta published in September 2024 for the first time combine recommendations for both diseases in a joint guideline document. The consolidation of PAD and aorta guidelines follows a holistic approach, which underlines the entirety of the arterial vascular system. This aim is underlined by a specifically introduced recommendation to take the entirety of the circulatory system into account in patients with vascular diseases. The focus in the current ESC guideline document is on a multidisciplinary, patient-centered management of PAD and diseases of the aorta, whereby the prevention and follow-up of patients after therapeutic interventions are emphasized. In PAD the document highlights exercise training and the procedure for patients with chronic wounds as well as risk stratification and hereditary diseases of the aorta.

## Einleitung

Die neuen Leitlinien der European Society of Cardiology (ESC) zum Management der peripheren arteriellen Krankheit und von Aortenerkrankungen („peripheral arterial and aortic diseases“, PAAD) beinhalten einige Neuerungen, die im Folgenden zusammengefasst werden sollen [1]. Bemerkenswert ist die Zusammenlegung von 2 zuvor separat geführten Leitliniendokumenten (ESC-Leitlinie zu Aortenerkrankungen von 2014 und ESC-Leitlinie zur peripheren arteriellen Verschlusskrankheit [pAVK] von 2017) zu einem Dokument [2, 3].

Im aktuellen ESC-Leitliniendokument wird auf die zunehmende Komplexität von Patienten mit pAVK und Aortenerkrankungen hingewiesen, und es werden ein ganzheitlicher Zugang sowie Multidisziplinarität im Patientenmanagement gefordert [1].

## Was ist neu in der Nomenklatur?

Eine weitere Neuerung zeigt sich bei der Nomenklatur. Im 2017 publizierten ESC-Leitliniendokument zur pAVK wurde die Bezeichnung „periphere arterielle Erkrankung“ („peripheral arterial disease“, PAD) als übergeordneter Begriff für sämtliche peripheren, nichtkoronaren Gefäßerkrankungen definiert [3]. Unter diesem Überbegriff wurden atherosklerotische Erkrankungen der Karotiden und Vertebralarterien, der Arterien der oberen Extremitäten, der Mesenterialarterien, der Nierenarterien sowie der Arterien der unteren Extremitäten („lower extremity artery disease“, LEAD) zusammengefasst. In der aktuellen ESC-Leitlinie 2024 wird der 2017 eingeführte Begriff LEAD nicht mehr verwendet, und die pAVK der unteren Extremität wird, wie schon vor der früheren Leitlinie, als PAD bezeichnet [1].

## Definition von Therapiezielen

Wie schon in der ESC-Leitlinie zur pAVK von 2017 wird im aktuellen Leitliniendokument die bei pAVK vorkommende Koprävalenz von koronarer und zerebrovaskulärer Atherosklerose unterstrichen, was das hohe Risiko koronarer und zerebrovaskulärer Ereignisse bei Patienten mit pAVK erklärt. Für das Management der pAVK wurden folglich als Therapieziele die Reduktion des Risikos von MACE („major adverse cardiac events“), die Reduktion des Risikos von MALE („major adverse limb events“) und die Verbesserung der Lebensqualität festgelegt [1].

Bei Aortenerkrankungen stellen die Prävention, die Screeningmaßnahmen und die rechtzeitige Initiierung der Therapie wesentliche Maßnahmen zur Reduktion der Morbidität und Mortalität dar.

## Empfehlungen zur Diagnose und Klassifizierung der pAVK

In der aktuellen ESC-Leitlinie wird empfohlen, bei Patienten ab 65 Jahre, insbesondere bei Vorliegen von kardiovaskulären Risikofaktoren, ein pAVK-Screening in Erwägung zu ziehen [1, 4, 5]. Bei Patienten mit 2 oder mehr kardiovaskulären Risikofaktoren kann ein pAVK-Screening unabhängig vom Alter erwogen werden [1, 6, 7].

**Abb. 1:**
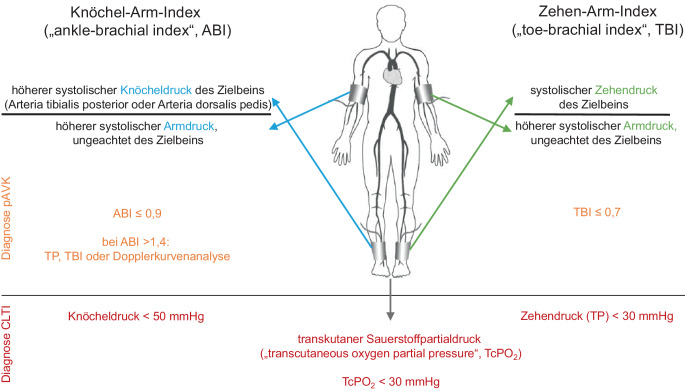
Diagnostik bei Verdacht auf periphere arterielle Verschlusskrankheit (*pAVK*) mit der Ermittlung des Köchel-Arm-Index („ankle-brachial Index“, *ABI*), des Zehen-Arm-Index („toe-brachial index“, *TBI*) und des transkutanen Sauerstoffpartialdrucks (*TcPO*_*2*_); Durchführung der Untersuchung in Liegeposition der Patienten (*TP* „toe pressure“, *CTLI* „chronic limb-threatening ischemia“). (Mod. nach [1])

Als Screeningmethode wird die Ermittlung des Köchel-Arm-Index („ankle-brachial index“, ABI) empfohlen. Ein ABI von 0,9 oder weniger kann dabei als diagnostisches Kriterium für das Vorliegen einer pAVK gewertet werden [8–10]. Bei Patienten mit Diabetes mellitus oder chronischer Nierenfunktionseinschränkung kann es infolge einer Mediasklerose zur Messung falsch-hoher Knöcheldruckwerte und folglich zur Ermittlung falsch-hoher ABI-Werte (> 1,4) kommen. In diesem Fall besteht eine I/B-Empfehlung, alternativ zum ABI die Messung des Zehendrucks, die Ermittlung des Zehen-Arm-Index („toe-brachial index“) oder Dopplerkurvenanalysen einzusetzen ([8, 11–14]; Abb. 1).

Je nach klinischer Präsentation werden unterschiedliche pAVK-Stadien unterschieden (Fontaine- oder Rutherford-Klassifikation). Im aktuellen ESC-Leitliniendokument wird eine Gruppierung in „asymptomatische pAVK“ (entspricht dem Fontaine-Stadium I), „symptomatische (belastungsabhängige) pAVK“ (entspricht den Fontaine-Stadien IIa und IIb) und „chronische beinbedrohende Ischämie“ („chronic limb-threatening ischemia“ [CLTI]; entspricht Fontaine-Stadium III und IV) vorgeschlagen (Tab. 1).

**Tab. 1:** Kategorisierung der peripheren arteriellen Verschlusskrankheit (*pAVK*). (Mod. nach [1])

Klinische Charakteristik	Fontaine-Klassifikation	
	Stadium	Klinische Präsentation
*Asymptomatische pAVK*	*I*	Keine durch pAVK bedingten Beschwerden
*Symptomatische (belastungsabhängige) pAVK*	*IIa*	Claudicatio intermittens mit subjektiv geringer Einschränkung
	*IIb*	Claudicatio intermittens mit subjektiv deutlicher Einschränkung
*CTLI*	*III*	Ischämischer Ruheschmerz
	*IV*	Ischämische Ulzera, Nekrosen oder Gangrän

*CTLI* „chronic limb-threatening ischemia“

## Einordnung von pAVK-Syndromen

Um der trotz vereinfachter Klassifikation für pAVK-Patienten bestehenden Vielfalt von klinischen Erscheinungsbildern nachzukommen, führt das aktuelle ESC-Leitliniendokument den Begriff der pAVK-Syndrome ein. Der Begriff „pAVK-Syndrom“ beschreibt das mögliche Vorliegen chronischer Wunden in unterschiedlichen klinischen Stadien der pAVK [1]. Ziel dieser Begrifflichkeit ist, das Vorhandensein einer chronischen Wunde bei pAVK-Patienten vom Vorliegen einer Minderperfusion, wie bei Vorliegen einer CLTI, zu entkoppeln. Um Wunden standardisiert zu charakterisieren und den Einfluss von Minderperfusion, Infektion und Wundausmaß zu graduieren, wird die Verwendung der WIfI(„wound, ischemia and foot infection“)-Klassifikation empfohlen [15, 16].

Im Behandlungsalgorithmus für pAVK-Syndrome wird empfohlen, ein Gefäßteam in die Entscheidungsfindung bezüglich der besten Therapieoptionen einzubeziehen.

Für Patienten mit symptomatischer (belastungsabhängiger) pAVK empfehlen die aktuellen ESC-Leitlinien ein supervidiertes Gehtraining (Klasse I, Evidenzgrad A; [17–21]). Das Gehtraining sollte bevorzugt an einer Gesundheitseinrichtung (3-mal pro Woche, 30 min pro Einheit, über eine Periode von ≥ 3 Monaten) stattfinden. Sollte das Gehtraining an einer Gesundheitseinrichtung nicht verfügbar sein, wird ein strukturiertes heimbasiertes Gehtraining mit Supervision (Kontrollvisiten oder Telefonate und Trainingsdokumentation mit Logbüchern) empfohlen [22].

Einen weiteren Schwerpunkt setzt die aktuelle ESC-Leitlinie auf die Nachuntersuchung der Patienten mit pAVK nach Therapieinitiierung. Im Rahmen der klinischen Evaluation soll neben dem kardiovaskulären Risikoprofil und dem klinischen Stadium der pAVK die Lebensqualität der Patienten standardisiert erhoben und dokumentiert werden [1].

Bei symptomatischer (belastungsabhängiger) pAVK besteht bei fehlender Besserung der Lebensqualität eine IIb/B-Empfehlung für die Planung eines revaskularisierenden Eingriffs [1, 23]. Bei endovaskulären Eingriffen wird femoropopliteal der Einsatz medikamentenbeschichteter Ballone oder Stents zur Reduktion des Rezidivrisikos nach erfolgreicher Revaskularisation empfohlen [1]. Bei langstreckigen femoropoplitealen Verschlüssen sollte bei guter Venenverfügbarkeit und niedrigem operativen Risiko eine chirurgische Venenbypassanlage in Erwägung gezogen werden [1].

Bei Vorliegen einer CLTI (Tab. 1) besteht in der ESC-Leitlinie eine klare Empfehlung für eine zeitnahe Revaskularisation zum Extremitätenerhalt [1]. In diesem Zusammenhang wird bei CLTI eine Bildgebung der gesamten betroffenen Extremität empfohlen, um den Eingriff planen zu können [24].

Zur Reduktion des Risikos ischämischer Ereignisse und zur Vermeidung eines erhöhten Blutungsrisikos wird ein individuelles Vorgehen in der antithrombotischen Therapie empfohlen. In diesem Zusammenhang sollten die klinische Präsentation („limb presentation“), das jeweilige Blutungsrisiko und die ggf. bestehende Notwendigkeit einer Antikoagulation aufgrund einer Komorbidität (z. B. Vorhofflimmern) berücksichtigt werden. Hervorgehoben wird in der aktuellen ESC-Leitlinie bei symptomatischer pAVK mit niedrigem Blutungsrisiko die „dual-pathway inhibition“ mit Acetylsalicylsäure (ASS) und niedrig dosiertem Rivaroxaban (1-mal täglich ASS 100 mg plus 2‑mal täglich Rivaroxaban 2,5 mg; [25–29]).

## Multimodaler Therapieansatz bei PAVK und Aortenerkrankungen

Das Management von Patienten mit pAVK und aortalen Erkrankungen ist charakterisiert durch nichtmedikamentöse und medikamentöse Maßnahmen, sowie – bei ausgewählten Patienten – durch endovaskuläre oder chirurgische Interventionen.

Hinsichtlich Lebensstilmodifikation wird im aktuellen ESC-Leitliniendokument erstmals eine Ernährungsempfehlung für Patienten mit pAVK und Aortenerkrankungen abgegeben (ausgewogene mediterrane ballaststoff- und flavonoidreiche Ernährung mit hohem Anteil an Hülsenfrüchten; [30–35]). Zusätzlich werden die Patientenaufklärung und verhaltensmodifizierende Interventionen mit dem Ziel der Nikotinkarenz empfohlen, wobei unterstützend vorübergehend E‑Zigaretten zum Einsatz gebracht werden können [36–38].

Bei der medikamentösen Therapie spielen neben der oben genannten antithrombotischen Medikation die antihypertensive und die lipidsenkende Therapie sowie die Behandlung des Diabetes eine wesentliche Rolle, um das kardiovaskuläre Risiko zu senken.

Bei pAVK besteht eine Klasse-I/Evidenzgrad-A-Empfehlung zu einer Statintherapie [39–42]. Das LDL(„low-density lipoprotein“)-Cholesterin(LDL-C)-Ziel liegt bei weniger als 55 mg/dl, und das Ausgangs-LDL‑C sollte um mehr als 50 % im Vergleich zum Ausgangswert gesenkt werden [43–46]. Wird das LDL-C-Ziel mit einer maximal tolerierten Statindosis nicht erreicht, sollte Ezitimib ergänzt werden [47]. Reicht diese Kombination weiterhin nicht aus, um das LDL-C-Ziel zu erreichen, findet sich im aktuellen ESC-Leitliniendokument eine I/A-Empfehlung für den Einsatz eines PCSK9(Proproteinkonvertase Subtilisin/Kexin Typ 9)-Inhibitors [48, 49]. Bei Statinunverträglichkeit und fehlendem Erreichen des LDL-C-Ziels mit Ezitimib sollte Bempedoinsäure oder eine Kombination aus Bempedoinsäure mit einem PCSK9-Inhibitor zum Einsatz kommen [50].

Bei der Blutdrucktherapie besteht im aktuellen Leitliniendokument eine I/A-Empfehlung für ein systolisches Blutdruckziel von 120–129 mm Hg, wobei primär ACE(„angiotensin-converting enzyme“)-Hemmer oder Angiotensinrezeptorblocker zum Einsatz kommen sollten [51–55]. Es wird in 2 separaten Empfehlungen ein etwas liberaleres Blutdruckziel von weniger als 140/90 mm Hg für fragile Patienten mit pAVK und Aortenerkrankungen empfohlen (Alter ≥ 85 Jahre, Pflegebedürftigkeit, orthostatische Hypotension, Lebenserwartung < 3 Jahre; [1]). Die Empfehlungen für die antihypertensive Medikation wurden mit den zeitgleich veröffentlichten ESC-Leitlinien zum Management erhöhten Blutdrucks in Einklang gebracht [45].

Beim Management von Diabetes mellitus besteht für Patienten mit pAVK und Aortenerkrankungen ein HbA_1c_(Hämoglobin A_1c_)-Ziel von weniger als 7 % (Empfehlungsgrad I, Evidenzgrad A), wobei auch hier eine Individualisierung des HbA_1c_-Ziels in Abhängigkeit von den Komorbiditäten und der Lebenserwartung als Konsensusempfehlung ergänzt wurde [1]. In Analogie zu der vor 1 Jahr publizierten ESC-Leitlinie zu kardiovaskulären Erkrankungen bei Diabetes empfiehlt auch das aktuelle Leitliniendokument unabhängig vom HbA_1c_-Wert SGLT(„sodium-glucose linked transporter 2“)-Hemmer oder GLP(„glucagon-like peptide“)-1-Rezeptor-Agonisten mit nachgewiesenem kardiovaskulärem Nutzen [56–68].

## Empfehlungen zum Management atherosklerotischer Läsionen der supraaortalen Arterien, der Nieren- und der Viszeralarterien

### Karotis- und Subklaviastenosen

Das ESC-Leitliniendokument beinhaltet für Patienten mit 2 oder mehr kardiovaskulären Risikofaktoren eine IIb/C-Empfehlung für ein sonographisches Screening hinsichtlich einer Karotisstenose [1].

Für Patienten mit diagnostizierter Karotisstenose werden – in Abhängigkeit vom Fehlen oder Vorliegen einer zugehörigen neurologischen Symptomatik – 2 separate Behandlungsalgorithmen vorgeschlagen. Die zugrunde liegende Quantifizierung des Stenosegrades erfolgt primär sonographisch (PSV [„peak systolic velocity“]: 125–230 cm/s oder ≥ 180 cm/s oder ≥ 125 cm/s, gemeinsam mit dem Vorliegen einer Ratio der PSV aus der A. carotis interna und der PSV aus der A. carotis communis ≥ 2 als Kriterien für einen Stenosegrad von 50–69 %; PSV > 230 cm/s als Kriterium für einen Stenosegrad ≥ 70 %), wobei bei symptomatischer Karotisstenose eine weiterführende Bildgebung mittels Computertomographie-Angiographie (CTA) oder Magnetresonanzangiographie (MRA) durchgeführt werden sollte [69, 70]. Bei asymptomatischer wie auch bei symptomatischer Karotisstenose wurde in den aktuellen Empfehlungen die optimale medikamentöse Therapie (OMT) zu Beginn der jeweiligen Behandlungsalgorithmen hervorgehoben [1]. Eine klare I/A-Empfehlung für eine zeitnahe Revaskularisation besteht lediglich bei der symptomatischen Karotisstenose mit einem Stenosegrad von 70–99 % [71, 72]. Bei 50–69 % einer symptomatischen Karotisstenose kann eine Revaskularisation in Erwägung gezogen werden (Klasse-IIa/A-Empfehlung).

Bei asymptomatischer 60–99 %iger Karotisstenose wird empfohlen, eine Revaskularisation individuell, d. h. in Abhängigkeit vom jeweiligen Risikoprofil bzw. vom Eingriffsrisiko, in Erwägung zu ziehen (Klasse-IIb/B-Empfehlung; [1]). Die Revaskularisationsmethode (Karotisdesobliteration vs. Karotisstent) soll ebenso in Abhängigkeit von klinischen und anatomischen Gegebenheiten gewählt werden:

- **klinische Risikofaktoren:**
  Herzinsuffizienz (NYHA [New York Heart Association] III/IV), instabile Angina pectoris (CCS [Canadian Cardiovascular Society] III/IV), koronare Herzkrankheit mit Hauptstammläsion oder > Einkoronargefäßerkrankung mit 70 %iger Stenose, rezenter Myokardinfarkt (< 30 Tage), geplante Herzoperation (< 30 Tage), linksventrikuläre Ejektionsfraktion (LVEF) < 30 %, schwere Lungenerkrankung, schwere Nierenfunktionseinschränkung;
- **anatomische Risikofaktoren:**
  chirurgisch schwierig/nicht zugängliche Läsionen (Höhe Wirbelkörper C2 oder darüber, unter dem Schlüsselbein), ipsilaterale Strahlentherapie, immobilisierte Halswirbelsäule, kontralateraler Karotisverschluss (erhöhtes Schlaganfallsrisiko), kontralaterale Rekurrensparese, Tracheostoma.

Zum nicht-invasiven Screening hinsichtlich Subklaviastenosen werden bei Patienten mit pAVK oder aortalen Erkrankungen beidseitige Blutdruckmessungen empfohlen (Blutdruckdifferenz > 10–15 mm Hg hinweisgebend für eine Subklaviastenose; [1]). Bei Vorliegen einer symptomatischen Subklaviastenose besteht im ESC-Leitliniendokument eine IIa/B-Empfehlung für eine Revaskularisation, wobei ein endovaskulärer Zugang einem offen-chirurgischen Vorgehen vorgezogen werden sollte [73]. Eine routinemäßige Revaskularisation von asymptomatischen Subklaviastenosen wird nicht empfohlen [1].

### Nierenarterienstenose

Bei Verdacht auf das Vorliegen einer Nierenarterienstenose empfehlen die ESC-Leitlinien auch primär eine duplexsonographische Diagnostik (Kriterien: PSV ≥ 200 cm/s entspricht > 50 %iger Stenose, renoaortale Ratio der PSV ≥ 3,5 entspricht ≥ 60 %iger Stenose bzw. Seitendifferenz des intrarenalen Widerstandsindex ≥ 0,5; [1]). Bei inkonklusivem duplexsonographischen Befund oder Verdacht auf das Vorliegen einer Nierenarterienstenose besteht eine I/B-Indikation für eine weiterführende Bildgebung mittels CTA oder MRA.

Bei Vorliegen von Risikomarkern (rasch progredienter therapierefraktärer Hypertonus, rasche Verschlechterung der Nierenfunktion, hypertensives Lungenödem, Vorliegen einer Einzelniere) und erhaltener Nierenvitalität (Nierengröße > 8 cm, Nierenkortex > 0,5 cm, Albumin-Kreatinin-Ratio < 20 mg/mmol, Widerstandsindex < 0,8) wird nach OMT eine invasive katheterbasierte Abklärung mit ggf. Stentimplantation empfohlen [1]. Ähnliches gilt für bilaterale Nierenarterienstenosen und für Patienten mit fibromuskulärer Dysplasie, wobei bei Letzterer eine primäre Ballondilatation gegenüber einer primären Stentimplantation bevorzugt werden sollte. Von einer routinemäßigen Revaskularisation einer einseitigen Nierenarterienstenose ohne Vorliegen oben genannter Risikomarker wird abgeraten, ebenso bei Fehlen der Nierenvitalität (Empfehlungsgrad III, Evidenzgrad A; [74–79]).

### Stenosen der Mesenterialarterien

Bei Verdacht auf chronische atherosklerotische Stenosen der Mesenterialarterien empfiehlt das ESC-Leitliniendokument zur bildgebenden Diagnostik eine CTA [1]. Je nach bildgebendem Befund werden atherosklerotische Stenosen von der nichtokklusiven mesenterialen Ischämie (NOMI) und vom Ligamentum-arcuatum-Syndrom unterschieden. Unter dem Begriff NOMI wird eine mesenteriale Ischämie verstanden, welche sich nicht auf Verschlüsse großer Mesenterialarterien zurückführen lässt, sondern auf eine Minderperfusion in der mesenterialen Endstrombahn, häufig infolge einer Vasokonstriktion in diesem Bereich, wie z. B. bei schwerer Herzinsuffizienz. Das Ligamentum-arcuatum-Syndrom benennt ein Kompressionssyndrom des Truncus coeliacus durch das Ligamentum arcuatum des Zwerchfells, was folglich zu einer symptomatischen Perfusionsstörung führen kann.

Während eine Revaskularisation von asymptomatischen Stenosen abgelehnt wird (III/C), empfiehlt das aktuelle ESC-Leitliniendokument bei symptomatischen Stenosen die Einbindung eines Gefäßteams in den Entscheidungsprozess hinsichtlich der Revaskularisationsindikation und -methode.

## Empfehlungen zu Aortenerkrankungen

Bei Vorliegen atheromatöser Plaques infolge aortaler Atherosklerose werden von der ESC-Leitlinie – unabhängig von der Anamnese früherer embolischer Ereignisse – eine 1‑fache plättchenfunktionshemmende Therapie und eine lipidsenkende Therapie mit einem LDL-C-Ziel von weniger als 55 mg/dl empfohlen [1].

Hinsichtlich eines Screenings auf das Vorliegen eines abdominellen Aortenaneurysmas (AAA) empfiehlt die ESC-Leitlinie 2024 die Ultraschalldiagnostik bei Männern ab 65 Jahre mit Raucheranamnese und bei Männern ab 75 Jahre unabhängig vom Raucherstatus. Bei Frauen ab 75 Jahre wird ein AAA-Screening bei Vorliegen einer Raucher- oder Hypertonieanamnese empfohlen [1]. Für Familienmitglieder von Patienten mit AAA besteht in der Leitlinie eine Empfehlung für ein AAA-Screening ab einem Alter von 50 Jahren (Empfehlungsgrad I, Evidenzgrad C; [1]). Nach Diagnose eines AAA sollte ein duplexsonographisches Screening hinsichtlich femoropoplitealer Aneurysmen in Erwägung gezogen werden. Bei Männern besteht ab einem AAA-Durchmesser von mehr als 55 mm und bei Frauen ab einem AAA-Durchmesser von 50 mm eine Klasse-I/Evidenzgrad-A-Empfehlung für eine Sanierung eines AAA [80–83]. Wie im letzten Aortenleitliniendokument wird auch im aktuellen Papier bei einem AAA-Wachstum von 5 mm oder mehr in 6 Monaten oder von mehr als 10 mm in 1 Jahr empfohlen, eine AAA-Sanierung in Erwägung zu ziehen [1, 2, 81]. Bei sakkulären AAA wird empfohlen, eine AAA-Sanierung schon ab einem maximalen Querdurchmesser von 45 mm in Erwägung zu ziehen [84].

Wichtig ist der Zusatz, dass die AAA-Sanierung bei einer voraussichtlichen Lebenserwartung von unter 2 Jahren kritisch hinterfragt werden sollte. Außerdem besteht keine Indikation (III/C-Empfehlung), vor einer AAA-Sanierung routinemäßig eine koronarangiographische Abklärung oder routinemäßig eine koronare Revaskularisation durchzuführen [1].

Bei Diagnose einer thorakalen Aortendilatation wird eine echokardiographische Untersuchung der Aortenklappe empfohlen (I/B-Empfehlung; [1]). Zur Dokumentation des Baseline-Durchmessers sowie zur Beurteilung der aortalen Symmetrie sollte jedoch eine weiterführende Bildgebung mit kardialer Magnetresonanztomographie oder kardialer CT folgen [85–87].

Bei einer Dilatation der Aorta ascendens und trikuspider Aortenklappe wird eine operative Sanierung bei einem Aortendurchmesser oder bei einem Durchmesser der Aortenwurzel von 55 mm oder mehr empfohlen [88–90]. Neben den genannten Durchmesserangaben werden zusätzliche morphologische Kriterien in der Indikationsstellung zur aortalen Sanierung in Erwägung gezogen (Tab. 2; [1]). Eine operative Sanierung wird bei bikuspider Aortopathie bei einem Durchmesser von 55 mm oder mehr empfohlen. Bei bikuspider Aortopathie und Root-Phänotyp (aortale Dilatation mit tubulärem Durchmesser > Sinusdurchmesser) wird eine Sanierung bei einem Durchmesser von 50 mm oder mehr empfohlen [1].

**Tab. 2:** Rupturrisikofaktoren bei thorakoabdominellem Aortenaneurysma (zusätzlich zum Aortendurchmesser). (Mod. nach [1])

*Aortenwurzel und Aorta ascendens*	Körpergröße des Patienten	Jährliche Größenprogredienz des Aortendurchmessers	Obere Normalgrenzen des Aortendurchmessers nach Patientenalter
	Resistenter Bluthochdruck	Länge der Aorta	Aortenwurzelaneurysma vs. Aneurysma der Aorta ascendens
*Aorta thoracica*	Resistenter Bluthochdruck	Sakkuläres Aneurysma im Zusammenhang mit PAU	COPD
*Thorakoabdominelle Aorta*	Wachstumsrate der Aorta: Aorta ascendens und Aortenbogen ≥ 3 mm/Jahr DTAA ≥ 10 mm/Jahr (oder ≥ 5 mm/6 Monate) AAA ≥ 10 mm/Jahr (oder ≥ 5 mm/6 Monate)	Genetische Prädisposition	Symptome
*Radiologische Merkmale der AAA-Ruptur*	Retroperitoneales Hämatom	Thrombusspaltung	Paraortale Fettablagerung
	Halbmondzeichen („high crescent sign“)	Kontrastmittelextravasation	Diskontinuität der Verkalkung
	Tangentiale Kalzifizierung	„Draped aorta sign“	

*COPD* „chronic obstructive pulmonary disease“,* DTAA* „descending thoracic aortic aneurysm“, *PAU* penetrierendes Aortenulkus, *AAA* abdominelles Aortenaneurysma

Bei Aortenbogenaneurysma wird bei thorakalen Beschwerden und niedrigem oder intermediärem operativen Risiko eine offen-chirurgische Sanierung empfohlen (Klasse-I/Evidenzgrad-B-Empfehlung; [1]). Bei asymptomatischen Patienten sollte bei einem Durchmesser von 55 mm oder mehr eine chirurgische Sanierung erfolgen (Klasse-IIa/Evidenzgrad-B-Empfehlung; [2, 89, 91]).

Bei Aneurysma der deszendierenden thorakalen Aorta wird bei einem Durchmesser von 55 mm oder mehr eine Sanierung empfohlen (Klasse-I/Evidenzgrad-B-Empfehlung; [92, 93]).

**Abb. 2:**
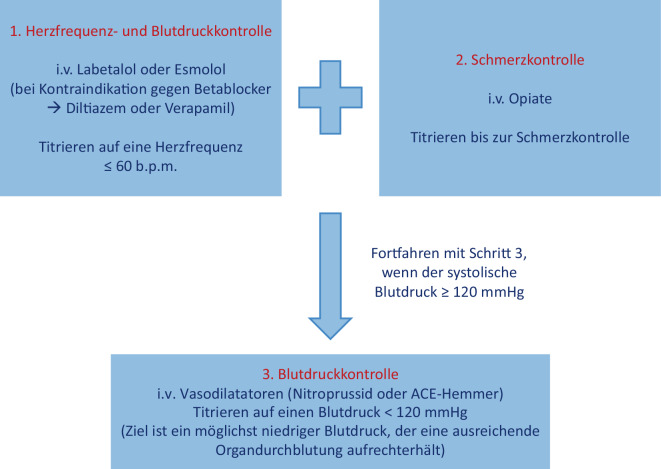
Medikamentöses Management bei akuten Aortensyndromen (*b.p.m.* „beats per minute“, *ACE* „angiotensin-converting enzyme“). (Mod. nach [1])

Präsentieren sich Patienten mit Verdacht auf ein akutes Aortensyndrom, wird ein multiparametrischer diagnostischer Algorithmus hervorgehoben [1]. Hierbei werden Hochrisikobedingungen (Marfan-Syndrom, Familienanamnese, bekannte Aortenklappenerkrankung, rezente aortale Manipulation, bekanntes Aortenaneurysma), Hochrisikoschmerzsymptome (Brust- oder abdomineller „reißender“ starker Schmerz mit plötzlichem Beginn) und Hochrisikountersuchungsergebnisse (hämodynamische Instabilität, peripheres Perfusionsdefizit, neurologisches Defizit, neues Strömungsgeräusch) evaluiert und entsprechend Punkte vergeben. Je nach Punktzahl in diesem Algorithmus kann eine Risikoeinstufung vorgenommen werden und die weitere diagnostische Abklärung erfolgen. Bei der Bildgebung mittels CT sollte eine EKG(Elektrokardiogramm)-getriggerte kardiale CT mit zusätzlicher Darstellung der gesamten Aorta erfolgen. Die medikamentöse Therapie orientiert sich an der Blutdruck‑, Herzfrequenz- und Schmerzkontrolle (Abb. 2); die Sanierung sollte an einem erfahrenen Aortengefäßzentrum erfolgen.

Bei penetrierenden Aortenulzera (PAU) wird bei Typ-A-PAU (Lokalisation in der Aorta ascendens) eine operative Sanierung empfohlen, während bei Typ-B-PAU (Lokalisation in der mittleren oder deszendierenden Aorta thoracalis) ein initial konservatives Vorgehen mit Imaging-Kontrollen empfohlen wird (Klasse-I/Evidenzgrad-C-Empfehlung; [1]). Bei komplizierten Typ-B-PAU oder unkompliziertem Typ-B-PAU mit Risikokonstellation wird eine endovaskuläre Versorgung empfohlen. Bei ausgewählten Patienten mit unkompliziertem Typ-A-PAU ohne Risikokonstellation und mit hohem operativen Risiko kann auch ein abwartendes Vorgehen gewählt werden [1].

## Genetische Aortenerkrankungen

Beim Management genetischer Erkrankungen mit Beteiligung der Aorta wird ein individualisiertes Vorgehen an einem Aortengefäßzentrum empfohlen. Die ESC-Leitlinie empfiehlt bei Patienten mit Aortenwurzel‑/Ascendensaneurysma oder thorakaler Aortendissektion das Einholen der Familienanamnese über zumindest 3 Generationen hinsichtlich thorakaler Aortenerkrankungen, plötzlichen Herztodes und peripherer oder intrakranieller Aneurysmen (Empfehlungsgrad I, Evidenzgrad B; [1, 94]). Bei Vorliegen eines Aortenwurzel‑/Ascendensaneurysmas oder einer thorakalen Aortendissektion mit Risikofaktoren für hereditäre Aortenerkrankungen werden eine genetische Beratung und ggf. Testung an einem Zentrum empfohlen.

Bei Patienten mit Marfan-Syndrom wird eine medikamentöse Therapie mit Betablockern oder Angiotensinrezeptorblockern in maximal tolerierter Dosierung empfohlen, um die Entwicklung einer Aortendilatation zu bremsen (Empfehlungsgrad I, Evidenzgrad A; [95, 96]).

Bei Patienten mit Loeys-Dietz-Syndrom werden im aktuellen ESC-Leitliniendokument Aortenwurzeldurchmessergrenzwerte (> 45 mm) für ein operatives Vorgehen angeführt [1].

## Fazit für die Praxis

- Der Schwerpunkt der ESC(European Society of Cardiology)-Leitlinien 2024 zur peripheren arteriellen Verschlusskrankheit (pPAVK) und zu Aortenerkrankungen liegt auf einem ganzheitlichen, gefäßmedizinischen Ansatz.
- Dieser Ansatz hebt die Prävention, die frühe Diagnose, ein individuelles patientenzentriertes Management und eine regelmäßige Nachsorge bei diesen Krankheitsbildern hervor und fordert die dafür erforderliche Multidisziplinarität im Patientenmanagement.
